# The bacterial *rhizobiome* of hyperaccumulators: future perspectives based on omics analysis and advanced microscopy

**DOI:** 10.3389/fpls.2014.00752

**Published:** 2015-01-07

**Authors:** Giovanna Visioli, Sara D'Egidio, Anna M. Sanangelantoni

**Affiliations:** Department of Life Sciences, University of ParmaParma, Italy

**Keywords:** hyperaccumulators, rhizosphere, endosphere, metals, omics, microscopy, phytoremediation

## Abstract

Hyperaccumulators are plants that can extract heavy metal ions from the soil and translocate those ions to the shoots, where they are sequestered and detoxified. Hyperaccumulation depends not only on the availability of mobilized metal ions in the soil, but also on the enhanced activity of metal transporters and metal chelators which may be provided by the plant or its associated microbes. The *rhizobiome* is captured by plant root exudates from the complex microbial community in the soil, and may colonize the root surface or infiltrate the root cortex. This community can increase the root surface area by inducing hairy root proliferation. It may also increase the solubility of metals in the rhizosphere and promote the uptake of soluble metals by the plant. The bacterial *rhizobiome*, a subset of specialized microorganisms that colonize the plant rhizosphere and endosphere, makes an important contribution to the hyperaccumulator phenotype. In this review, we discuss classic and more recent tools that are used to study the interactions between hyperaccumulators and the bacterial *rhizobiome*, and consider future perspectives based on the use of omics analysis and microscopy to study plant metabolism in the context of metal accumulation. Recent data suggest that metal-resistant bacteria isolated from the hyperaccumulator rhizosphere and endosphere could be useful in applications such as phytoextraction and phytoremediation, although more research is required to determine whether such properties can be transferred successfully to non-accumulator species.

## Hyperaccumulators and the *rhizobiome*

Hyperaccumulators are plants that accumulate metals and/or metalloids in their leaves at concentrations several orders of magnitude higher than the levels tolerated by other species. The hyperaccumulator phenotype has evolved in environments where restrictive growth conditions allow the adaptation of only a few plant species (Baker et al., 2000; Pollard et al., 2014). The root environment is a dynamic microsystem in which microbes, roots, and the soil interact, and the roots can gain access to soil nutrients and metals (Alford et al., 2010). Root system development, root morphology and chemotropism are all recognized as equally important for establishing the hyperaccumulator phenotype, although the mechanisms involved are still not fully understood (Moradi et al., 2009). Bacteria and fungi colonizing the rhizosphere (immediately surrounding the root) and the endosphere (compartments within the root) play an important role in the establishment of interactions between hyperaccumulators and the soil. These microbes tend to be metal tolerant and can promote plant growth in contaminated soils by several mechanisms: inducing the formation of hairy roots (thus increasing the root surface area), enhancing the solubility and uptake of metal ions and producing phytormons and metabolites (Rajkumar et al., 2012). The beneficial effects of metal-tolerant microbes have attracted attention because of their biotechnological applications in plant-based remediation strategies (Salt et al., 1995). The characterization of the hyperaccumulator *rhizobiome* is therefore needed to facilitate such applications (Mastretta et al., 2006; Rajkumar et al., 2009; Ma et al., 2011a; Sessitsch et al., 2013). In this review, we discuss classical and more recent omics-based methods that are used, and can be used, to study the interaction between hyperaccumulators and the bacterial *rhizobiome*, combined with advanced microscopy techniques for the visualization of microbe–host systems, emphasizing the potential applications of these microorganisms in phytotechnology.

## Culture-dependent vs. culture-independent methods

**Figure 1:**
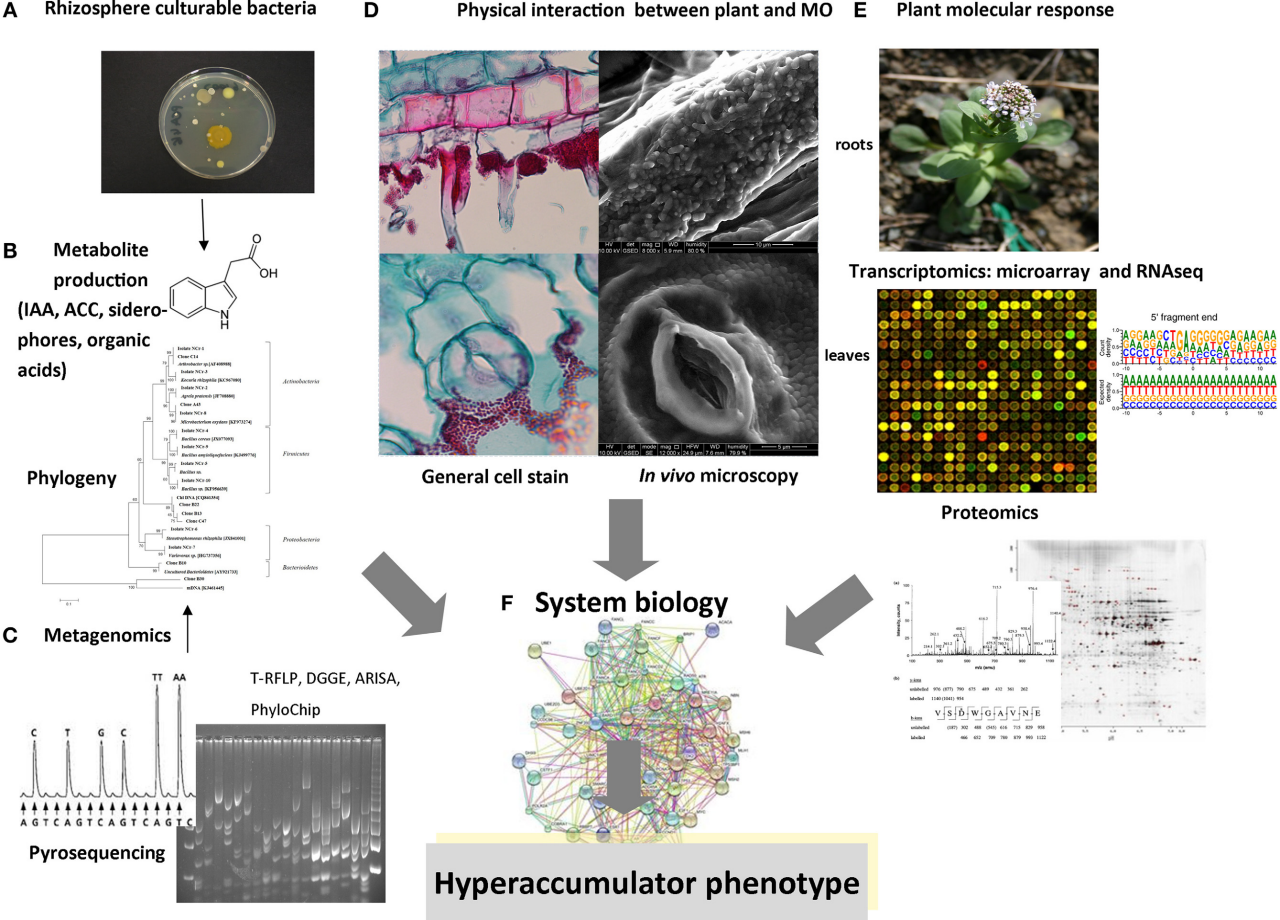
**Schematic representation of multidisciplinary approaches for the analysis of *rhizobiome*–hyperaccumulator interactions. (A)** Bacteria in the rhizosphere and endosphere that are amenable to laboratory cultivation can be analyzed for metal resistance and **(B)** for the production of metabolites. **(C)** Metagenomics involves the extraction of DNA from roots and rhizosphere compartments, followed by techniques such as 16*S* rDNA amplification, cloning, and sequencing, fingerprinting (RFLP analysis, DGGE, ARISA, PhyloChip), or direct next-generation sequencing. **(D)** Physical interactions between bacteria and plants can be visualized using advanced optical and electron microscopy methods following the inoculation of seeds or roots with bacteria. **(E)** Microarray and RNA-Seq technologies can be used to analyze bacterial gene expression following exposure to root exudates, or to compare root/shoot gene expression between plants cultivated in the presence and absence of bacteria. Proteomic analysis can also identify plant proteins that are modulated by the *rhizobiome* and characterize the roles of bacteria that promote the hyperaccumulator phenotype. **(F)** The goal of future studies will be to collect and correlate data from all these methods in a system biology approach to define the molecular basis of metal accumulation in plants.

The structure and diversity of microbial communities in the rhizosphere and endosphere of several plants (the *rhizobiome*) has been analyzed in detail at the molecular level in order to characterize the interaction between microbes and plants (Sørensen et al., 2009). However, less than 10% of hyperaccumulator species have been investigated (Alford et al., 2010). The principal approach to study bacteria in the hyperaccumulator *rhizobiome* was mainly based on traditional culture-dependent techniques (Table 1). These comprise fractionation protocols, in which roots are shaken in high ionic solutions to remove bacteria from soil particles and the rhizobacteria are collected by washing. Roots surfaces are then sterilized and sonicated to remove the epidermal cells and macerated for the extraction of endophytes. Serial dilutions are then prepared from the wash fraction (rhizobacteria) and the crushed roots (endophytes) and these are inoculated on rich agar medium supplemented with heavy metals. This allows bacterial strains showing high resistance to metals to be selected and tested for the production of metabolites such as siderophores, organic acids and phytohormones, which may be responsible for promoting root growth and solubilization of metals (Figures 1A,B). If the isolated bacterial strains are amenable to laboratory cultivation, they can be used to inoculate hyperaccumulator plants so as to optimize plant growth and metal extraction capacity. Alternatively, they can be used to inoculate higher-biomass non-accumulator plants to determine the transferability of metal tolerance traits, which would make those species more suitable for phytoremediation applications (Table 1).

**Table 1:** **Studies resuming recent literature about culture-dependent hyperaccumulators endophytes and rhizobacteria and their effects on plant growth and metal accumulation**.

**Hyperaccumulator host**	**Metal**	**Bacterial strains**	**Source**	**Methodology for bacteria characterization**	**Beneficial features and metabolite production**	**Effect on plant**	**References**
*Alyssum bertoloni*	Ni	*Microbacterium* sp.	Endosphere	Cultivation dependent technique ARDRA	Siderophore, high level of resistanace to heavy metals	Root colonizing ability	Barzanti et al., 2007
		*Arthrobacter* sp.					
		*Micrococcus* sp.					
		*Bacillus* sp.					
		*Paenibacillus* sp.					
		*Leifsonia* sp.					
		*Curtobacterium* sp.					
		*Pseudomonas* sp.					
*Alyssum murale*	Ni	*Microbacterium oxydans*	Rhizosphere	Cultivation dependent technique	Ni mobilization	↑Ni uptake	Abou-Shanab et al., 2003, 2006
		*Microbacterium liquefaciens*			Siderophore		
		*Microbacterium arabinogalactanolyticum*			P-solubilization		
*Alyssum pintodasilvae*	Ni	*Arthrobacter nicotinovorans* SA40	Rhizosphere (*Alyssum serpyllifolium)*	Cultivation dependent technique	ACCD, siderophore, IAA, Ni-solubilization	↑Ni uptake	Cabello-Conejo et al., 2014
						↑Plant biomass	
*Alyssum serpyllifolium*	Ni	*Arthrobacter* sp.	Rhizosphere	Cultivation dependent technique	Siderophore, IAA, P-solubilization organic acids	↑Ni uptake	Becerra-Castro et al., 2011, 2013
						↑Metal and mineral solubility	
				BOX-PCR fingerprinting		↑Plant biomass	
						↑Shoot nutrient concentrations	
*Arabidopsis halleri*	Cd-Zn	*Pseudomonas* sp.	Rhizosphere	Cultivation dependent technique	n.d.	↓Plant biomass	Farinati et al., 2009, 2011
		*Chryseobacterium* sp.				↓Cd Zn shoot content	
		*Tsukamurella* sp.				↑Chlorophyll content	
		*Escherichia coli*				↑Photosynthesis- and abiotic stress-related proteins	
		*Sphingomonas* sp.					
		*Curtobacterium* sp.				↓Plant defense-related proteins	
*Brassica napus*	Pb	*Microbacterium* sp.	Endosphere	Cultivation dependent technique	ACCD, siderophore, IAA	↑Root elongation	Sheng et al., 2008
		*Pseudomonas fluorescens*				↑Pb uptake and TF	
						↑Plant biomass	
	Cu	*Microbacterium* sp.	Rhizosphere of Cu-tolerant plants *(Commelina communis, Rumex acetosa, Kummerowia striata, and Bidens bipinnata)*	Cultivation dependent technique and DGGE	ACCD, siderophore, IAA, P-solubilization	↑Root elongation	He et al., 2010
		*Pseudomonas chlororaphis*					
		*Arthrobacter* sp.					
		*Microbacterium lactium*					
		*Azotobacter vinelandii*					
	Cd Pb Zn	*Enterobacter* sp.	Rhizosphere of *Polygonum pubescens*	Cultivation dependent technique	ACCD, siderophore, IAA, P-solubilization	↑Cd, Zn, Pb uptake	Jing et al., 2014
		*Klebsiella* sp.					
	Cu	*Acinetobacter* sp.	Endosphere of *Elsholtzia splendens* and *Commelina communis*	Cultivation dependent technique and molecular cloning	ACCD, siderophore, IAA, arginine decarboxylase	↑Cu uptake and TF	Sun et al., 2010
		*Moraxella* sp.				↑d.w.	
		*Serratia* sp.					
		*Herbaspirillum* sp.					
		*Bukholderia* sp.					
		*Paracoccus* sp.					
		*Sphingomonas* sp.					
		*Exiguobacterium* sp.					
		*Bacillus* sp.					
		*Arthrobacter* sp.					
		*Microbacterium* sp.					
		*Micrococcus* sp.					
	Cd-Pb-Zn	*Rahnella* (JN6)	Endosphere of *Polygonum pubescens*	Cultivation dependent technique	ACCD, siderophore, IAA, P-solubilization	↑Cd Pb Zn uptake	He et al., 2013
						↑Cd Pb Zn tolerance and mobilization	
						↑Plant biomass root interior colonization	
*Brassica juncea*	Cd	*Rhodococcus* sp.	Endosphere	Cultivation dependent technique	ACCD, siderophore, IAA	↑Root elongation	Belimov et al., 2005
		*Flavobacterium* sp.					
		*Variovorax paradoxus*					
	Ni	*Pseudomonas* sp.	Rhizosphere of *A.serpyllifolium, Astragalus incanus* and *Phleums phleoides*	Cultivation dependent technique	ACCD, siderophore, IAA, P-solubilization	↑Plant biomass	Ma et al., 2009a,b
		*Psychrobacter* sp.				↑Ni uptake	
		*Bacillus* sp.				↑Root and shoot elongation	
*Chenopodium ambrosioides*	Pb-Zn	*Pseudomonas* sp.	Rhizosphere	Cultivation dependent technique and molecular cloning	Siderophore, IAA	↑Plant biomass	Zhang et al., 2012
		*Exiguobacterium* sp.				↑Pb uptake	
		*Arthrobacter* sp.					
		*Flavobacterium* sp.					
		*Chryseobacterium* sp.					
		*Paenibacillus* sp.					
		*Sphingobacterium* sp.					
		*Comamonas* sp.					
*Noccaea caerulescens*	Ni	*Bacillus* sp.	Endosphere	Cultivation dependent technique and molecular cloning	ACCD, siderophore, IAA	↑Root elongation	Visioli et al., 2014
		*Microbacterium* sp.				↑Ni TF	
		*Arthrobacter* sp.				↑Plant biomass	
		*Kocuria rhizophila*					
	Zn	*Microbacterium saperdae*	Rhizosphere	Cultivation dependent technique	n.d.	↑Zn solubility in soil	Whiting et al., 2001
		*Pseudomonas monteilii*					
		*Enterobacter cancerogenus*					
	Zn	*Sphingomonas* sp.	Rhizosphere and Endosphere	Cultivation dependent technique	n.d.	n.d.	Lodewyckx et al., 2002
		*Metylobacterium* sp.					
		*Aureobacterium esteraromaticum*					
		*Nocardioides* sp.					
		*Matsuebacter chitosanotabidus*					
		*Variovorax* sp.					
		*Rhodococcus* sp.					
*Pteris multifida*	As	*Bacillus* sp.	Endosphere	Cultivation dependent technique	IAA	↑Arsenic tolerance	Zhu et al., 2014
		*Paenibacillus* sp.					
		*Lysinibacillus* sp.					
		*Massilia* sp.					
		*Micrococcus* sp.					
		*Brevundimonas* sp.					
		*Paracoccus* sp.					
		*Curtobacterium* sp.					
		*Roseomonas* sp.					
		*Staphylococcus* sp.					
		*Sphingomonas* sp.					
		*Microbacterium* sp.					
*Petris vittata*	As	*Naxibacter* sp.	Rhizosphere	Cultivation dependent technique	n.d.	n.d.	Huang et al., 2010
		*Pseudomonas* sp.					
		*Bacillus* sp.					
		*Acinetobacter* sp.					
		*Caryophenon* sp.					
	As	*Bacillus* sp.	Endosphere	Cultivation dependent technique	IAA	↑Arsenic tolerance	Zhu et al., 2014
		*Paenibacillus* sp.					
	As	*Pseudomonas* sp.	Rhizosphere	Cultivation dependent technique	Siderophore	↑As solubilization	Ghosh et al., 2011
		*Comamonas* sp.				↑As uptake	
		*Stenotrophomonas* sp.				↑Root d.w	
*Salix caprea*	Zn-Cd	*Bradyrhizobium* sp.	Rhizosphere	Cultivation dependent technique	ACCD, siderophore, IAA production of metal-mobilizing metabolites	Rhizosphere isolate: ↓Metal uptake in roots	Kuffner et al., 2010
		β-proteobacteria				Endophyte isolate: ↑TF	
		Actinobacteria				↑Zn-Cd mobilization plant interior colonization	
		Chlorobi					
		*Burkholderia* sp.	Endosphere				
		*Sphingomonas* sp.					
		*Metylobacterium* sp.					
		Actinobacteria					
*Sedum alfredii*	Cd-Zn	*Burkholderia cepacia*	Rhizosphere	Cultivation dependent technique	n.d.	↑Plant biomass under Zn treatment	Li et al., 2007
						↑Root biomass under Cd treatment	
						↑Cd-Zn uptake and TF	
						↑P uptake	
		*Burkholderia* sp.	Endosphere		ACCD, IAA, biofilm formation, root colonization		Zhang et al., 2013
		*Sphingomonas* sp.					
		*Variovorax* sp.					
	Zn-Cd Pb-Cu	5 unidentified bacterial strains	Rhizosphere of *S. alfredii* treated with multi-metals	Cultivation dependent technique	n.d.	↑Root elongation	Xiong et al., 2008
						↑Chlorophyll content	
						↑Zn-Cd- Pb-Cu uptake	
						↑Plant Biomass	
						↑Chlorophyll, N and P content	
						↑Heavy metals tollerance	
	Zn	*Pseudomonas* sp.	Endosphere	Cultivation dependent technique	Siderophore, IAA, Nitrogen fixation, P and Zn-solubilization	↑Zn solubilization and bioavailability	Long et al., 2011, 2013
		*Bacillus pumilus*				↑Zn uptake	
		*Stenotrophomonas* sp.				↑Plant biomass	
		*Acinetobacter* sp.					
*Solanum nigrum*	Cd	*Arthrobacter* sp.	Endosphere	Cultivation dependent technique	ACCD, siderophore, IAA, P-solubilization	↑Plant biomass	Luo et al., 2011
		*Microbacterium* sp.				↑Cd uptake, BCF and TF	
		*Bacillus* sp.					
		*Flavobacterium* sp.					
		*Agrobacterium* sp.					
		*Serratia* sp.					
		*Chryseobacterium* sp.					
		*Sphingomonas* sp.					
		*Pseudomonas* sp.					
	Cd	*Serratia nematodiphila*	Endosphere	Cultivation dependent technique	ACCD, siderophore, IAA, P-solubilization	↑Plant biomass	Chen et al., 2010
		*Enterobacter* sp.				↑Cd uptake and TF	
		*Acinetobacter* sp.					
		*Pseudomonas* sp.	Endosphere	Cultivation dependent technique	Siderophore, biosurfactants, organic acid	↑Shoot d.w.	Chen et al., 2014a,b
						↑Cd uptake, BCF and TF	
						↑Fe and P uptake	
						↑Heavy metals uptake	
*Thlaspi goesingense*	Ni	*Bacillus* sp.	Rhizosphere and endosphere	Cultivation dependent technique and RFLP	ACCD, siderophore	n.d.	Idris et al., 2004
		*Blastococcus* sp.					
		*Propionibacterium acnes*					
		*Flavobacterium* sp.					
		*Desulfitobacterium metallireductans*					
		Methylobacterium mesophilicum					
		Methylobacterium extorquens					
		*Sphingomonas* sp.					
		*Curtobacterium* sp.					
		*Plantibacter flavus*					
		*Rhodococcus* sp.					

Information in this table is arranged in alphabetical order of host plant binomial name. Abbreviations: ↑, Increase; ↓, Decrease; ACCD, 1-aminocyclopropane-1-carboxylate deaminase; IAA, indole-3-acetic acid; n.d., not detected; d.w., dry weight; DGGE, denaturing gradient gel electrophoresis; RFLP, restriction fragment length polymorphism; T-RFLP, terminal restriction fragment length polymorphism; TRF, terminal restriction fragment; TF, translocation factor; BCF, bioconcentration factor.

Thus far, bacteria found to be associated with hyperaccumulators using cultivation-dependent methods mainly comprise the Gram-positive genera *Arthrobacter*, *Microbacterium*, *Bacillus* and *Curtobacterium*, and the Gram-negative genera *Pseudomonas, Sphingomonas*, and *Variovorax* (Table 1). These genera have been found in both the rhizosphere and the endosphere of hyperaccumulators regardless the specific metal composition of the soil. Other genera appear to be more prevalent in soils containing specific metals, e.g., *Chrysobacterium* and *Burkholderia* tend to be associated with Cd/Zn hyperaccumulators. The data presented in Table 1, summarizing recent literature about endophytes and rhizobacteria of hyperaccumulators, show that the transfer of such bacteria to non-accumulator plants has in many cases boosted the accumulation of plant biomass, root proliferation, metal uptake and metal translocation to aerial parts of the plant, although some species benefit from only a subset of these effects. A complete absence of beneficial effects was observed in only two cases. These results highlight both the general positive impact of the bacteria *rhizobiome* on plant growth and its specific influence on metal accumulation.

Metal resistance is a common feature of the *rhizobiome* in metalliferous soils but not among the bacteria inhabiting normal soils. Furthermore, endophytic bacteria from the inner tissues of hyperaccumulators may have adapted to withstand higher metal concentrations than the rhizobacteria (Idris et al., 2004). The presence of endophytic populations in different organs and tissues (e.g., roots, stems, and leaves) may also explain their variable levels of metal tolerance (Barzanti et al., 2007; Long et al., 2011; Ma et al., 2011b; He et al., 2013).

Many bacteria present in the rhizosphere and endosphere cannot be cultivated under laboratory conditions but are nevertheless important mediators of plant–soil interactions. Approximately 99% of microorganisms in the biosphere have never been recovered by standard cultivation techniques and it is necessary to study these species using cultivation-independent approaches. Methods based on direct isolation and analysis of proteins, lipids and nucleic acids from environmental samples have been developed to reveal structure diversity, functions and dynamics of microbial communities without cultivation (Kirk et al., 2004; Rastogi and Sani, 2011) but, so far, only few of these methods have been applied to study bacterial communities associated with hyperaccumulators, which are not suitable for laboratory cultivation.

The extraction of total genomic DNA from the rhizosphere and/or endosphere of hyperaccumulators followed by the preparation of 16S rDNA clone libraries provides sufficient material for an initial survey of microbial diversity and facilitates the identification of novel taxa. This method has been used to study bacteria associated to *Solanum nigrum* roots and copper and lead tolerant/resistant plants (Sun et al., 2010; Zhang et al., 2012; Chen et al., 2014a,b). The sequencing of clone libraries based on amplified 16S rRNA genes offers the highest phylogenetic resolution but microbial diversity can be underestimated because many clones are required to document the richness present in rhizosphere samples. One additional bottleneck is the co-amplification of 16S rDNA from plant organelles, although this can be overcome using primers specific for bacterial rDNA (Chelius and Triplett, 2001). These primers exclude chloroplast DNA and give a larger mitochondrial PCR product (Idris et al., 2004). Genetic fingerprinting techniques (Figure 1C) have been used as an alternative to clone libraries and these are more sensitive because they are applied directly to the extracted genomic DNA. Denaturing gradient gel electrophoresis (DGGE), terminal restriction fragment length polymorphism (T-RFLP) analysis and automated ribosomal intergenic spacer analysis (ARISA) have been applied to hyperaccumulators (He et al., 2010; Gupta et al., 2014). Although they do not always allow the immediate taxonomic identification of all species in the community, they can track the dominant members in a complex environment and allow the comparison of bacterial communities in different settings (Rastogi and Sani, 2011).

The PhyloChip16S rDNA microarray could also provide a high-throughput and comprehensive overview of microbial communities in environmental samples, but cross-hybridization is a major limitation and this method cannot detect novel taxa because only sequences represented on the chip are interrogated (Sanguin et al., 2006; Rastogi and Sani, 2011).

More recently, the emergence of next-generation sequencing (NGS) technologies such as the Roche/454, Illumina/Solexa, Life/APG, and HeliScope/Helicos BioSciences platforms (Figure 1C) has revolutionized environmental microbiology and made it possible to resolve complex microbiomes with greater accuracy and associate the diversity of microbial communities with their niche functions (Knief, 2014). Notably, short-read methods such as pyrosequencing have dramatically reduced the time and cost of microbial whole-genome sequencing projects, and have also facilitated the ultra-high-throughput sequencing of hypervariable regions of 16S rRNA genes with 2–3 orders of magnitude greater coverage than Sanger sequencing. Even short hypervariable sequences (100–350 bp) provide sufficient phylogenetic information for taxonomic profiling, so multiple environmental samples can be combined in a single run, and the reads can be parsed using their assigned nucleotide barcode, which is added to the templates by PCR (Metzker, 2010). NGS technologies have been used to study the *rhizobiome* of *Arabidopsis thaliana* (Bulgarelli et al., 2012; Lundberg et al., 2012), *Populus deltoides* (Gottel et al., 2011), *Lactuca sativa* (Schreiter et al., 2014), and *Zea mays* (Peiffer et al., 2013) but this approach has not yet been used to characterize the microbial community associated with hyperaccumulators (Knief, 2014). However, NGS has been used to analyze the bacterial community in soils polluted with heavy metals and to determine the impact of heavy metal contamination on the composition of the community (Berg et al., 2012; Gołębiewski et al., 2014). It has also been used to determine the impact of genotype and soil type on the microbiome (Peiffer et al., 2013; Ge et al., 2014; Zachow et al., 2014). Metagenomics therefore appears to be an ideal approach to investigate the diversity and ecology of the hyperaccumulator *rhizobiome*.

## *In situ* analysis of plant–rhizobiome interactions

Over the last two decades there have been significant developments in the methods used for the localization and *in situ* visualization of microbes inside and around plant roots, matching the advances in molecular microbiology described above (Figure 1D). Specific bacterial populations can be detected and localized using fluorescence microscopy or electron microscopy combined with tagging techniques (Sørensen et al., 2009). There have been few studies of the physical plant–microbe interactions around the roots of hyperaccumulators, but the role of specific bacterial strains can be investigated by monitoring the colonization and survival of inoculums under real environmental conditions using *in situ* microscopy (Figure 1D). In this context, environmental scanning electron microscopy (ESEM) is a powerful system that allows the observation of biological specimens *in situ* without sample preparation (Stabentheiner et al., 2010). The ESEM specimen chamber operates slightly above the saturation vapor pressure of water. Under such conditions, water remains in the liquid phase and hydrated biological samples can be observed without fixation and dehydration, which is normally required for conventional scanning electron microscopy. As an example, this method has been used to investigate the physical association between the roots and shoots of the Ni hyperaccumulator *Noccaea caerulescens* and Ni-resistant endophytic bacteria and rhizobacteria of the genera *Microbacterium* and *Arthrobacter* (Visioli et al., 2014).

In the model plant species *A. thaliana*, conventional epifluorescence microscopy and confocal laser scanning microscopy has been used to localize bacteria in the rhizosphere and within plant tissues (Compant et al., 2005; Bulgarelli et al., 2012; Cardinale, 2014). The abundance and composition of the bacterial community can be investigated using strain-specific fluorescent antibodies, fluorescence *in situ* hybridization (FISH) against rRNA or mRNA targets, and more recent methods such as catalyzed reporter deposition (CARD)-FISH (Pernthaler et al., 2002; Bulgarelli et al., 2012; Lundberg et al., 2012).

In addition, the localization and functional analysis of specific bacteria within plant tissues can be achieved by expressing reporter genes encoding fluorescent marker proteins such as green fluorescent protein (GFP), which can be integrated directly into the bacterial chromosome or into a plasmid that is subsequently introduced into the bacteria (Sørensen et al., 2009). One of the advantages of the reporter gene strategy is that the marker protein can be expressed constitutively or induced by external factors such as the presence/concentration of specific chemicals, including metals (Ramos et al., 2002; Rothballer et al., 2005). Gram-positive Actinobacteria such as the genera *Arthrobacter* and *Microbacterium* are present in the hyperaccumulator *rhizobiome* and have been shown to promote plant growth and metal absorption. Although these bacteria are usually recalcitrant to transformation, a *Microbacterium* strain was recently transformed with a GFP probe before inoculation onto sugarcane plants to study plant–microbe interactions (Lin et al., 2012). The success of this approach suggests that a similar strategy could be used to investigate microbial interactions with hyperaccumulators.

## The plant growth-promoting capacity of the *rhizobiome*

As shown in Table 1, the rhizobacteria and endophytes associated with hyperaccumulators often promote the growth of their host plants and increase their capacity for metal accumulation. This could be achieved by the production of siderophores and carboxylic acids, or the solubilization of phosphates to increase the mobility of metals in the rhizosphere (Li et al., 2007; Ma et al., 2009b; Cabello-Conejo et al., 2014), thus enhancing the accumulation of metals by roots and shoots (Sheng et al., 2008; Sun et al., 2010; Luo et al., 2011; He et al., 2013). Plant-associated microbes can also promote growth indirectly by protecting their hosts against pathogens, or directly by producing phytohormones (such as indole acetic acid, abscisic acid and gibberellic acid) or by secreting enzymes such as 1-aminocyclopropane-1-carboxylic acid deaminase which, reducing ethylene levels, allows plant growth and resistance to environmental stresses. Classical culture-based methods are often used to test bacteria for the production of siderophores and other secondary metabolites (Sheng et al., 2008). However, more sensitive approaches based on gas chromatography and mass spectrometry can be used to detect metabolites produced by bacteria *in situ* without sample preparation, e.g., nanospray desorption ionization (nano-DESI) (Traxler and Kolter, 2012; Watrous et al., 2012). These are highly sensitive techniques that rapidly determine the metabolites present in a sample and they help to identify and quantify new compounds produced by bacteria which can be beneficial for plant growth and metal accumulation in contaminated soils.

## Transcriptomics and proteomics

The composition and genetic capabilities of the hyperaccumulator *rhizobiome* can be characterized by metagenomics analysis as described above, but this does not reveal the ability of microbes to respond to particular stimuli, and the functions of the majority of microbial species that inhabit intercellular spaces in the root are still poorly understood (Hirsch and Mauchline, 2012). Transcriptomics and proteomics can be useful in this regard because both approaches show how the complementary functions of plants and their associated microbial communities are expressed (Figure 1E). The study of such interactions not only contributes to our understanding of plant–microbe relationships but also facilitates the development of novel strategies to promote phytoremediation.

Microarrays provide a useful platform to analyze the transcriptomes of plants and the microbes that inhabit the rhizosphere, although they can only monitor the expression of genes that are represented on the array (Mark et al., 2005; Fan et al., 2012; Kwak et al., 2012). In contrast, NGS can be used to sequence entire transcriptomes with unparalleled accuracy, resolution and throughput, and with no limitations in terms of sequence coverage. This approach is known as RNA-Seq, and has already been used to study of abiotic stress responses (including exposure to metals) in bacteria isolated from the rhizosphere or contaminated soils, and for comparative studies of gene expression in the roots of hyperaccumulators adapted to grow in different metalliferous soils (Maynaud et al., 2013; Halimaa et al., 2014; López-Leal et al., 2014). This is an ideal approach for the analysis of plant–*rhizobiome* interactions at the transcriptomic level. RNA-Seq and microarray analysis can also be complemented with comparative proteomics to determine the proteins that are synthesized or modified during such interactions (DalCorso et al., 2013; Visioli and Marmiroli, 2013). For example, comparative proteomics was used to look for proteins expressed by the Cd/Zn accumulator *Arabidopsis halleri* in the presence or absence of specific Cd-resistant microbes or the entire autochthonous *rhizobiome*. The presence of the *rhizobiome* correlated with the accumulation of both Cd and Zn in the shoots, and this involved the upregulation of proteins involved in photosynthesis and the Calvin cycle, whereas defense proteins and antioxidant enzymes were down-regulated (Farinati et al., 2009, 2011). The *A. halleri* proteome responded differently to the presence of the total *rhizobiome* compared to selected bacterial strains, indicating that the *rhizobiome* as a community is required for the most efficient hyperaccumulation phenotype (Farinati et al., 2011).

In the same manner that metagenomics can be used to sample the genetic potential of the microbial community, metaproteomics can be used to sample the proteins present among complex environmental consortia in extreme environments such as metalliferous soils. However, several empirical, technical, computational, and experimental design challenges remain to be addressed, including the development of efficient techniques for protein extraction from soils and subsequent sample preparation (Leary et al., 2013). Several organic compounds in the soil (e.g., humic acids) can interfere with protein identification, and the samples are prone to degradation so the amount of available metaproteomic data is currently limited (Leary et al., 2013). Even so, the presence (or absence) of specific microbial proteins will eventually be useful as an indicator for positive interactions between the plant root and soil microbes, allowing the prediction of hyperaccumulator phenotypes.

## Conclusions

The composition of the bacterial *rhizobiome* coupled with the genomic, transcriptomic, and proteomic analysis of plant–microbe interactions may help us to understand in more detail the associations between hyperaccumulators and the surrounding bacterial communities of the endosphere and rhizosphere. It will be interesting to compare the *rhizobiome* of different facultative metallophytes, such as *N. caerulescens* adapted to grow in different metalliferous and non-metalliferous soils (Pollard et al., 2014), because this will help to isolate the bacteria that contribute to the hyperaccumulator phenotype. However, the rhizosphere is a dynamic environment with the community undergoing rapid spatiotemporal changes in response to external factors. The metabolic profiling of microbial colonies by *in situ* mass spectrometry (Traxler and Kolter, 2012) should therefore be integrated with omics-based profiling methods in a systems biology approach (Figure 1F) to facilitate the investigation of interactions between the *rhizobiome* and hyperaccumulator plants, thus providing an advanced toolkit for phytotechnology applications.

### Conflict of interest statement

The authors declare that the research was conducted in the absence of any commercial or financial relationships that could be construed as a potential conflict of interest.
